# Dissecting the Genetic Basis of Lateral and Central Spikelet Development and Grain Traits in *Intermedium*-Spike Barley (*Hordeum vulgare* Convar. *Intermedium*)

**DOI:** 10.3390/plants9121655

**Published:** 2020-11-26

**Authors:** Helmy M. Youssef, Mohamed Allam, Faiza Boussora, Axel Himmelbach, Sara G. Milner, Martin Mascher, Thorsten Schnurbusch

**Affiliations:** 1 Leibniz Institute of Plant Genetics and Crop Plant Research (IPK), Corrensstr. 3, OT Gatersleben, 06466 Seeland, Germany; mohamed.allam@studenti.unitus.it (M.A.); boussora@ipk-gatersleben.de (F.B.); himmelba@ipk-gatersleben.de (A.H.); milner@ipk-gatersleben.de (S.G.M.); mascher@ipk-gatersleben.de (M.M.); 2 Faculty of Agriculture, Cairo University, Giza 12613, Egypt; 3 Institute of Agricultural and Nutritional Sciences, Faculty of Natural Sciences III, Martin Luther University Halle-Wittenberg, 06120 Halle, Germany; 4 Faculty of Agriculture, Assuit University, Assuit 71526, Egypt; 5 Institute of Arid Lands of Medenine, Route du Djorf km 22.5, Médénine 4100, Tunisia

**Keywords:** *intermedium* barley, spikelet development, fertility, central spikelet, lateral spikelet, GWAS

## Abstract

Barley (*Hordeum vulgare* L.) is one of the major grain crops worldwide and considered as a model plant for temperate cereals. One of the barley row-type groups, named *intermedium-*barley, was used in our previous study where we reported that other genetic loci rather than *vrs1* and *Int-c* could play a role in lateral spikelet development and even in setting grains. To continue this work, we used phenotypic and genotypic data of 254 *intermedium*-spike barley accessions aimed at dissecting the genetic basis of development and grain traits of lateral and central spikelet using genome wide association (GWAS) analysis. After genotypic data filtering, 8,653 single-nucleotide polymorphism (SNPs) were used for GWAS analysis. A total of 169 significant associations were identified and we focused only on the subset of associations that exceeded the *p* < 10^−4^ threshold. Thirty-three highly significant marker-trait-associations (MTAs), represented in 28 different SNPs on all seven chromosomes for the central and/or lateral spikelet traits; such as kernel length, width, area, weight, unfilled spikelet and 1000-kernel weight, were detected. Highly significant associated markers were anchored physically using barley genome sequencing to identify candidate genes to either contain the SNPs or the closest gene to the SNP position. The results showed that 12 MTAs were specific for lateral spikelet traits, nine MTAs were specific for central spikelet traits and seven MTAs for both central and lateral traits. All together, the GWAS and candidate gene results support our hypothesis that lateral spikelet development could be regulated by loci different from those regulating central spikelet development.

## 1. Introduction

Barley (*Hordeum vulgare* L.) is one of the most highly produced and consumed grains in the world, classified as the fourth major cereal crop globally [1]. It is an excellent experimental model for other cereals, due to its simple inbreeding and diploid genome. The barley inflorescence (called spike) consists of three single-flowered spikelets—one central and two lateral spikelets per rachis node—that confer unique row-type identity to barley spikes. The main distinction among cultivars is made based on the number of kernel rows, i.e., two-rowed and six-rowed barley spikes. In two-rowed barley only the central spikelet is fertile, resulting in a single grain; whereas there are three grains at each node in six-rowed barleys [2]. Domestication evolution has influenced morphological and physiological features of crops [3,4] constituting the domestication syndrome, for which crops depend on humans for their growth and reproduction. Disabling sterility in the lateral florets seems a straightforward way to triple the number of grains at each rachis node, that was the route of six-rowed barley to arose from the ancestral two-rowed barley. The *intermedium*-barley might be a transition form from two- to six-rowed barley.

There are more than 400,000 accessions of barley in worldwide gene banks [5] that are considered as an excellent genetic resource to be efficiently utilized in barley breeding programs. These accessions differ in their genetic constitution. The genetic regulation of crop plant fitness (the mutant × genotype interaction) across diverse environments [6] is under the focus of recent research. In addition, the high-quality barley reference genome sequence assembly became available in 2017 [7] and increased the interest of barley as an important model crop plant that now can be used to understand the genetic basis of barley (root, shoot, spike and spikelet) development. One of these genetic resources is known as *intermedium*-spike barley (*Hordeum vulgare* convar. *intermedium* (Körn.) Mansf. (syn. *H. intermedium* Körn.) that exhibits various degrees of lateral spikelet fertility and grain development, an intermediate form between two- and six-rowed types [8]. It might therefore provide an excellent opportunity to study the genetic basis controlling central and lateral spikelet fertility separately.

There are five row-type genes identified and characterised in barley, *Six-rowed spike 1* (*Vrs1*) encodes a homeodomain leucine zipper class I transcriptional activator [9,10] that inhibits the fertility of lateral spikelets. Functional mutations in VRS1 are sufficient to generate a full six-rowed spike phenotype. Meanwhile *VRS2*, *VRS3*, *VRS4* and *VRS5*/*INTERMEDIUM*-*SPIKE C* (*INT-C*) encode homologues of SHORT INTERNODES (SHI), putative Jumonji C-type H3K9me2/me3 demethylase, LATERAL ORGAN BOUNDARY (LOB) and TEOSINTE BRANCHED1/CYCLOIDEA/PROLIFERATING CELL NUCLEAR ANTIGEN FACTOR 1 (TB1), respectively [11,12,13,14,15,16]. The *int-c* locus, located on chromosome 4HS [12,17], has been reported to control the fertility of lateral spikelets and accordingly the row-type identity.

In our previous work [18], we re-sequenced the entire *Vrs1*, *Vrs4* and *Int-c* genes in the *intermedium* barley collection. The results showed that some of the *intermedium* accessions showed a complete six-rowed phenotype while carrying the wildtype two-rowed allele of *Vrs1* [9] in combination with the six-rowed allele of *int-c* that is considered as a modifier of lateral spikelet development [12]. Additionally, we found that the *Int-c.a* allele was not able to overpower the suppression of lateral florets in the genomic background of wild barleys and two-rowed-like forms of *intermedium* barley accessions that carried known functional *Vrs4* alleles [13] and the *Vrs1.b* allele [18]. These results suggested that other genetic loci or allelic combinations could play a role in lateral spikelet development and even in setting grains.

The rapid development of the genomic tools in DNA sequencing revolutionized the way to genetically improve important traits (e.g., yield traits) to bridge the gap between the world production and demands. One of the recent applications of next generation sequencing (NGS) is the genotyping by sequencing (GBS) approach that provides a huge amount of high-quality genome-wide genetic markers (single nucleotide polymorphisms, SNPs). These SNPs are suitable for studying diversity and marker-trait associations [19,20] and accelerate genetic breeding programs in crops [20]. Many powerful statistical genetics methods were also proposed to identify alleles controlling target traits. The associations between genotype and phenotype are dependent on linkage disequilibrium (LD), the non-random association of alleles, being broken down by many generations of recombination. A genome-wide association study (GWAS) is one of these useful methods that can be successfully applied to identify candidate genes for many important traits in barley as it tests the association between SNP markers and the phenotype of a target trait [21,22,23].

Here, we aimed to (1) better understand the genetic basis controlling spikelet development and growth. Moreover, we sought to (2) identify novel gene(s)/loci that could play a role in lateral spikelet development and even setting grains in the *intermedium* barley collection. Association mapping analyses have been used to identify SNP markers associated with heading and flowering time in barley (see, e.g., [24,25]). However, the genetic variation of spike morphology and its components is less studied. In this study, we are the first in barley to dissect the genetic basis of development and grain traits of lateral and central spikelet separately. Fourteen spikelet morphology and fertility-related traits were scored for central and lateral spikelets distinctly, and then phenotypic-genotypic association analysis was performed. Further quantitative trait loci (QTLs) analysis identified candidate genes in this study that will help to propose a genetic network (among novel and already known genes) underlying spikelet fertility traits in barley.

## 2. Results

### 2.1. Phenotypic Data Analysis

**Figure 1:**
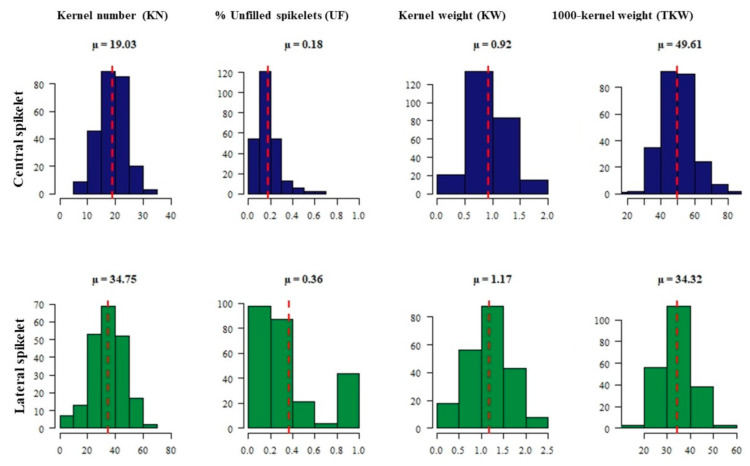
The phenotype’s frequency distribution histogram of kernel number (KN), percentage (%) of unfilled spikelets (UF), kernel weight (KW) and 1000-kernel weight (TKW) for central (blue color) and lateral (green color) spikelets among 254 *intermedium*-spike barley accessions.

**Figure 2:**
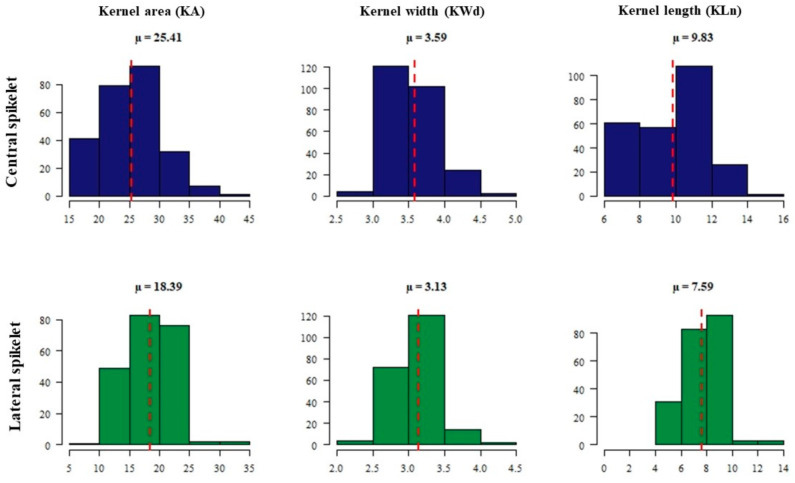
The phenotype’s frequency distribution histogram of kernel area (KA), kernel width (KWd), kernel length (KLn) and spikelet awn length (SAL) for central (blue color) and lateral (green color) spikelets among 254 *intermedium*-spike barley accessions.

Phenotypic variation was observed based on the average of the four biological replicates of each accession among the 254 *intermedium*-spike barley accessions for all traits. Frequency distributions of all accessions for the investigated traits are presented in Figure 1 and Figure 2.

The *intermedium*-spike barley collection showed different fertility degrees for lateral spikelets, with a range from 0%, i.e., completely infertile (two-rowed like), to 100% fertile (six-rowed like), with a mean value of 36%. However, only an average of 18% infertility was noticed for central spikelets. As expected, a higher number of kernels (KN) and lower 1000-kernel weight (TKW) of lateral spikelets were observed. The mean value for KN per spike of central spikelets was 19.03, while it was 34.75 for lateral spikelets. The mean value for TKW of central spikelets was about 50 g, while for lateral spikelets it was 34.32 g (Figure 1).

The average of kernel area (KA) of central spikelets was 25.41 mm^2^, with a range from 17 to 42 mm^2^. While, KA of lateral spikelets (18.39 mm^2^) was smaller by about 27.6%, with a range from 9.7 to 33.78 mm^2^. The mean values of kernel width (KWd) and length (KLn) were 3.59 and 9.83 mm^2^, respectively, of central spikelets. However, KWd and KLn of lateral spikelets were smaller by about 12.8% and 22.4%, respectively (Figure 2).

### 2.2. SNP Data and Population Structure Analysis

A total of 8,653 high quality SNPs distributed on the seven chromosomes (Supplementary Table S2) were selected for further analyses. The highest number of SNPs (17%, 1,458 of 8,653) was located on chromosome 2H and covered from nucleotide position 30,562 bp to 765,158,691 bp of the chromosome, while chromosome 4H contained the least number of SNPs (10%, 905 of 8653) and covered from 54,127 bp to 646,290,210 bp of the chromosome. The average number of SNPs/Mb was the lowest (7.4) on chromosome 1H and the highest (10.2) on chromosome 7H.

The population structure in the panel of *intermedium* barley was analysed using 8653 SNPs and a model-based approach in STRUCTURE program. The number of subpopulation (K) was plotted against the ΔK calculated and the peak of the broken line graph was observed at K = 6 (Supplementary Materials), which indicates that the most appropriate number of clusters are represented by six sub-groups in this population.

**Figure 3:**
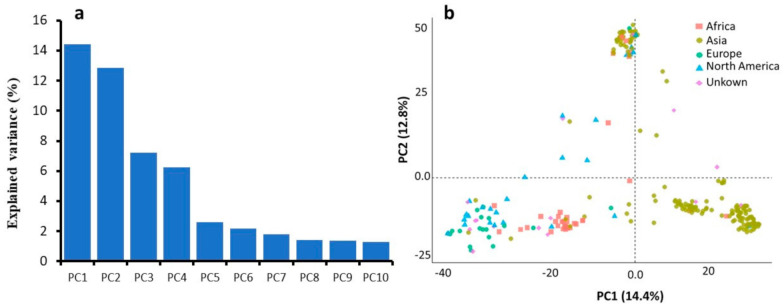
Plots for the principal components (PCs). (**a**) The scree plot shows the variance explained by the first 10 PCs. (**b**) The distribution of the 254 *intermedium*-spike barley lines in PC1 vs. PC2 (‘continents’ used as covariate).

A scree plot generated to visualize the fraction of variance explained by each of the 10 principle components, showed that PC1 and PC2 represented the highest proportion of the total variance, that together described about 27% of the population stratification (Figure 3a). PC1 explained about 14.4%, and PC2 explained about 12.8% of the total variance. The results of the PCA highlighted different sub-populations could be explained by geographical differences between accessions with few exceptions (Figure 3b).

### 2.3. GWAS Analysis and Candidate Genes

**Figure 4:**
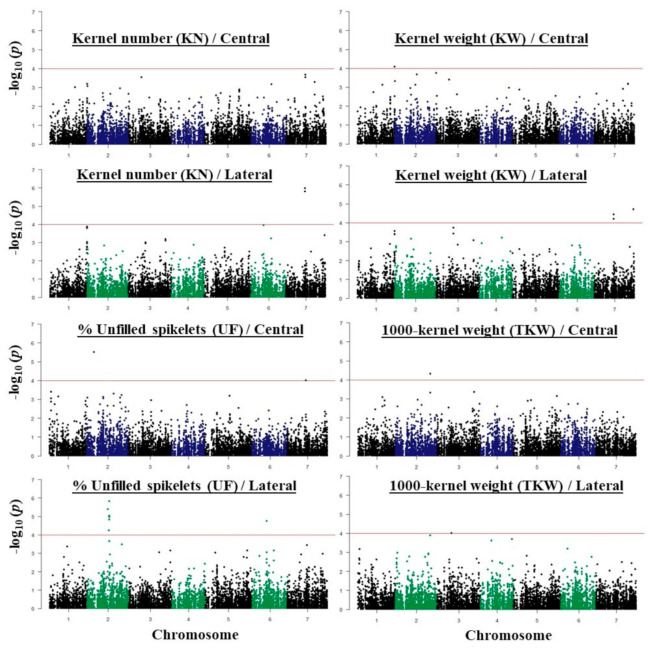
Manhattan plots of genome wide association (GWAS) conducted on kernel number (KN), percentage (%) of unfilled spikelets (UF), kernel weight (KW), and 1000-kernel weight (TKW) for central spikelets and lateral spikelets.

**Figure 5:**
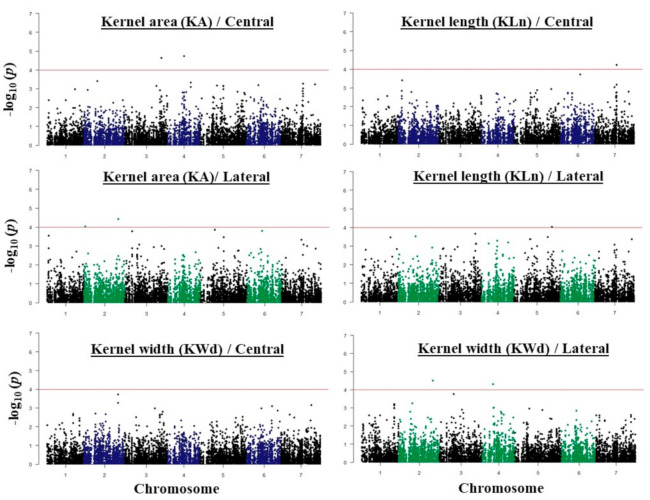
Manhattan plots of GWAS conducted on KA, KWd, and KLn, for central spikelets and lateral spikelets.

The results of a GWAS conducted for fertility and kernel traits of the *intermedium*-spike barley are presented in Figure 4 and Figure 5. The highest number of associations was found on chromosome 2H (41 associations) and the lowest was on chromosome 4H and 6H (13 associations). In this study, we focused only on the subset of 33 highly significant associations represented in 28 different SNPs that exceeded the *p* < 10*^−^*^4^ threshold (Table 1 and Table 2).

The significant marker-trait association (MTA) was detected on chromosome 1H, at 556,903,593 pb (m1_556903593) for KW and KN of central spikelets only. Interestingly we found that this SNP is located in the Early flowering 3 (ELF3) gene, in the exon at 45 bp from the start codon (Table 1). Another two highly significant MTAs were detected on chromosome 7H (m7_168762979 and m7_168763009) for KN of both central and lateral spikelets. These two markers were also found in KW of lateral spikelets only. At this position we found that these two SNPs are located in the Sec14p-like gene (Table 1 and Table 2). Another highly significant association was detected for KW of lateral spikelets and also located on chromosome 7H (m7_651215909). However, this marker was only identified for KN of lateral spikelets (Table 2). For UF, ten significant associations were detected on chromosome 2H: for central spikelets at 35,441,939 bp, while located in the range of 647,258,179 to 654,165,739 bp for lateral spikelets. From the candidate genes at latter position expansin B3, elongation factor G, aldehyde dehydrogenase family 3 member F1 and multiprotein bridging factor 1A were found only for lateral spikelets (Table 2). Interestingly, in this region the Vrs1 gene is co-located (chr2H:652,031,295 to 652,032,562) (Figure 4). Two other MTAs were found for UF, on chromosome 7H at 109,046,266 bp for central spikelets, and on chromosome 6H at 115,087,828 bp for lateral spikelets.

On chromosome 2H, the SNPs m2_727927744, m2_727927750 and m2_727927781 representing the same genomic region were detected for TKW and KWd of central and lateral spikelets and KA of lateral spikelets. However, another region was found on chromosome 3H (m3_661788536, m3_661788539, m3_661788540 and m3_661788542), associated with TKW and KA, but only for central spikelets, while m3_208358229 was detected for TKW, KA, and KWd, but only for lateral spikelets. On chromosome 4H at 26,351,423 bp, another MTA was detected for TKW and KWd for lateral spikelets. On chromosome 7H, marker (m7_548419008) was detected for KA of both central and lateral spikelets, and KLn of lateral spikelets only.

## 3. Discussion

In this study, we focused on a better understanding of the spikelet development and its effects on barley grain production. Yield is a complex agronomic trait, kernel number per spike and kernel weight and size are key factors determining final barley yield. Although some QTLs related to yield traits have been identified, such as grain length [26,27,28,29,30], TKW [29,30,31,32,33], grain width [26,27,29,30,32,34,35], grain area [26,29,34] on almost all seven chromosomes, not many genes affecting yield and spike architecture have been identified so far [2,9,12,36].

The current study is the first report exploring the genetic basis of natural variation in separated central and lateral spikelets of *intermedium*-spike barleys for morphological and fertility-related traits along the barley inflorescence using GWAS. We phenotyped mature spikes from 254 *intermedium*-spike barley accessions and found 169 significant MTAs position on all the seven chromosomes that showed associations with lateral and/or central spikelets traits. Here we focused our attention only on the 28 highest significant ones (Table 1 and Table 2) out of which 12 MTAs positions were detected for the lateral spikelets traits, nine MTAs for the central spikelets traits and seven MTAs positions for both central and lateral traits. From all of these SNP positions, only in a single case the well-known row-type gene *Vrs1* is co-located on Chr. 2H at position chr2H:652,031,295–652,032,562. Provided that most *intermedium* barley carry the functional two-rowed allele at *vrs1* and the six-rowed allele at *Int-c* [18], we assume that allelic variation is mainly fixed for both genes in our panel. Given that variation might be present but in low frequency at both genes, obtaining significant peaks from those genes appears less likely. We therefore assume that the peak on 2 HL, close to the *Vrs1* gene, is most likely related to other genes in LD. Nevertheless, these results support our previous finding that different loci, i.e., other than *vrs1* and *Int-c*, may control lateral and/or central spikelet development in this *intermedium* barley collection [18].

**Figure 6:**
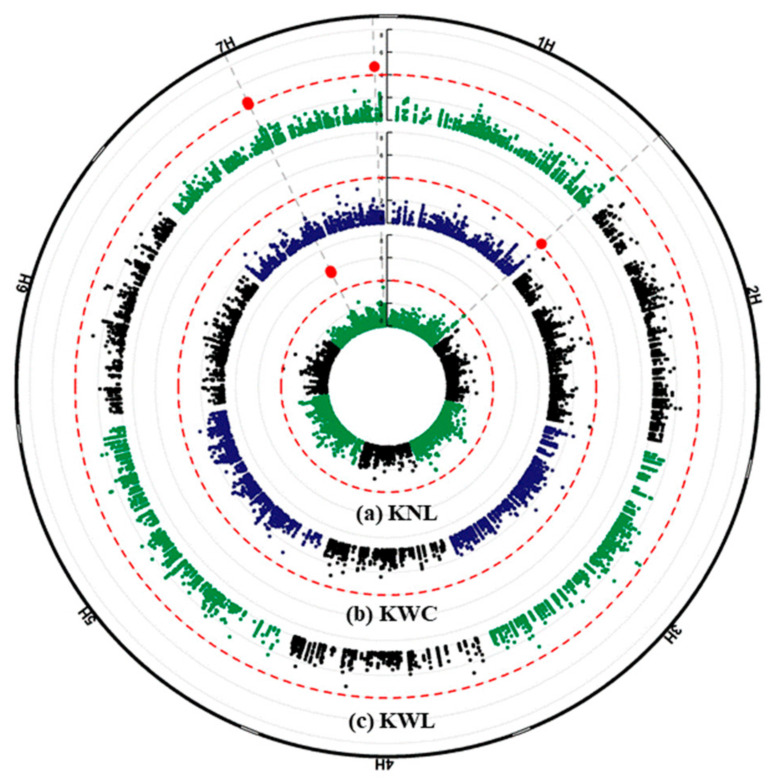
Circular Manhattan plots of genome-wide association scans for central (blue) and lateral (green) spikelets for (**a**) Kernel number (KN), (**b**) and (**c**) kernel weight (KW). The significance level of marker-trait associations (−log *p* values) is represented by the vertical scale bar. Individual chromosomes are represented on the outer circle and separated from eachD other by white borders. −log *p* values (*p* < 10^−4^) are indicated by dashed circles. Genomic regions of detected QTL on the respective chromosomes are colored in red (outer circle).

**Figure 7:**
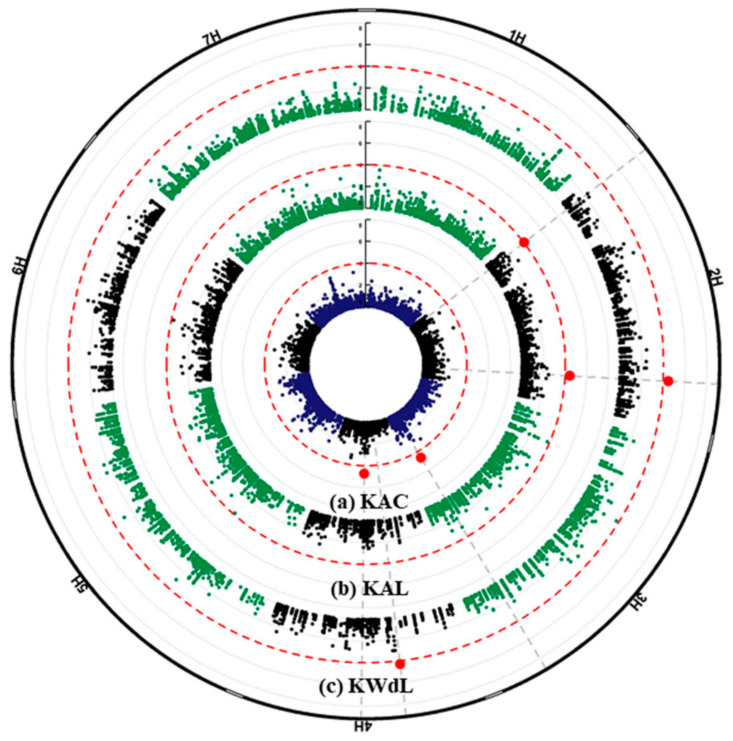
Circular Manhattan plots of genome-wide association scans for central (blue) and lateral (green) spikelets for (**a**) and (**b**) kernel area (KA), and (**c**) kernel width (KWd). The significance level of marker-trait associations (−log *p* values) is represented by the vertical scale bar. Individual chromosomes are represented on the outer circle and separated from each other by white borders. −log *p* values (*p* < 10^−4^) are indicated by dashed circles. Genomic regions of detected QTL on the respective chromosomes are colored in red (outer circle).

The thirteen MTAs detected for the lateral spikelets are located on chromosomes 2H to 7H. One of the interesting ones is located on chromosome 2H between 649,489,024 and 654,165,739 nucleotide positions for the %UF containing approx. ~160 HC genes. Interestingly, we found in this region *multiprotein bridging factor 1A* gene that carried three of the SNPs (m2_654165703, m2_654165718 and m2_ 654165739). Tsuda et al. (2004) [37] reported that *multiprotein bridging factor 1* (*MBF1*) in Arabidopsis functions as a transcriptional co-activator that mediates transcriptional activation by bridging between an activator and a TATA-box binding protein (TBP). Annotations of genes located on the other positions on chromosome 2H encode three different proteins; *Expansin B3* at m2_647258179, *Elongation factor G (EF-G)* at m2_651372029 and *Aldehyde dehydrogenase family 3member F1* at m2_653986096. All of these genes have unknown effects on the lateral spikelet development of barley. For lateral spikelets, an MTA at nucleotide position (208,358,229) on chromosome 3H was detected for KWd, KA and TKW, and another MTAs at nucleotide position (26,351,423) on chromosome 4H was detected but only for the KWd and TKW. Another MTAs on chromosome 2H (positions: m2_727927744 and m2_727927750) were detected for the KWd and KA for lateral spikelets (Table 2, Figure 6) as well as MTAs on chromosome 7H at positions (m7_168762979 and m7_168763009) for the KN and KW of lateral spikelets (Table 2, Figure 7). These results suggested a high correlation and effect of KWd KA and of KN, KW on TKW. The two main known row-type genes affecting barley grain size or weight that have been characterized so far are *Vrs1* located on chromosome 2 HL [9,38] and *Int-c* located on chromosome 4 HS [12] at a position 17,599,033–17,600,737 which is about 8.8 Mb distant from the detected SNP on 4 HS (m4_26351423). These results re-confirmed our previous findings that *Vrs1* and *Int-c* are not the only contributors affecting KWd, KA and TKW in this collection [18]. However, future work is required for validating the detected candidate regions by using either other GWAS panels or biparental mapping populations.

On chromosome 3H, we detected highly significant MTAs for KA and TKW but only for the central spikelet, suggesting that there are different loci in central spikelets playing a role in controlling these traits. Interestingly, the four detected SNPs (m3_661788536, m3_661788539, m3_661788540 and m3_661788542) are located in a single gene, a uridine-diphosphate (UDP)-glycosyltransferase superfamily protein. We found that three of these SNPs are causing changes in the amino acid sequence of this gene; m3_661788539 caused a serine (S) to arginine (R) change and m3_661788540 and m3_661788542 caused arginine (R) to glycine (G) changes at amino acid positions three and four, respectively. UDP-glycosyltransferases are enzymes that work on a large and diverse group of highly complex and diverse substrates such as different phytohormones (such as auxin, cytokinin and salicylic acid) and flavonoids as well as many others substrates to regulate plant growth and development, including biotic and abiotic resistance [39,40]. Their assigned function supports a putative role as a candidate gene in central spikelet development in barley; however, also in this case follow-up experiments are urgently required to verify this candidate.

**Figure 8:**
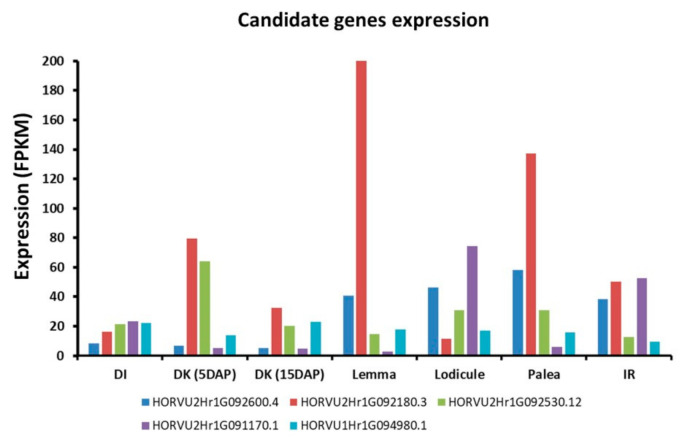
Expression analysis of the most important candidate genes: HORVU1Hr1G094980—*early flowering 3* (*ELF3*); HORVU2Hr1G092600—*multiprotein bridging factor 1A*; HORVU2Hr1G092180—*elongation factor G*; HORVU2Hr1G092530—*aldehyde dehydrogenase family 3 member F1*; HORVU2Hr1G091170—*expansin B3* in the developing inflorescence tissues; DI—developing inflorescences (1–1.5 cm); DK (5 DAP)—developing kernels (5 DAP); DK (15 DAP)—developing kernels (15 DAP); IR—inflorescences rachis (35 DAP); FPKM—fragments per kilobase of exon model per million reads mapped. (Source: Colmsee et al. 2015 [42]; https://apex.ipk-gatersleben.de/apex/f?p=284:10::::::).

Interestingly, we detected an MTA for central kernel number and kernel weight at 556,903,593 bp position on chromosome 1H. The annotation data showed that this SNP is located at *ELF3*. The *ELF3* gene is responsible for suppressing flowering under non-inductive photoperiods by regulating gibberellin production and *FLOWERING LOCUS T 1* (*FT1*) expression [41]. These results support our previous findings that phytohormones may have a key role during spikelet development in barley [14]. We also found MTA for two SNPs (m2_727927744, m2_727927750) on chromosome 2H for central and lateral spikelets associated with traits TKW, KA and KWd as well as MTA at a position 548,419,008 bp on chromosome 7H associated with KLn and KA for central and lateral spikelets. These results supported our hypothesis that KWd has an impact on KW, while KLn has an impact on KA. Notably, we found that all the candidate genes presented here are expressed in the developing inflorescence tissues (Figure 8).

All together, the GWAS and candidate gene results support our hypothesis that lateral spikelet development could be regulated by loci different from those regulating central spikelet development. Nevertheless, future experiments may have to reveal the nature of these insights in more detail.

## 4. Materials and Methods

### 4.1. Genetic Materials and Phenotyping

A set of 254 *intermedium*-spike barley accessions, classified based on lateral spikelet size as *intermedium*-spike barley [8] (*Hordeum vulgare* L. convar. *intermedium* (Körn.) Mansf.) from the German Federal ex situ Gene bank hosted at IPK-Gatersleben (Seeland, Germany), were used in this study (Supplementary Table S1). The association mapping panel is composed of spring and winter barley lines from different regions worldwide, originating from four continents (Asia, Africa, North America and Europe; Supplementary Materials) and 18 different countries (Supplementary Table S1 in Youssef et al. 2017a [18]).

Four plants from each accession of the *intermedium*-spike barleys were germinated in 96 wells trays and kept for 10–15 days till they reached the two- to three-leaf stage. At the three leaves stage, plantlets were vernalized for six weeks at 4 °C followed by hardening for one week under 12/12 h light conditions (day/night) and temperature 12 ± 2 °C day and night. Afterwards, each plant was transplanted into 14 cm diameter pot and grown in potting substrate (peatmoss fertilised with N|:|P|:|K, 14|:|16|:|18) in the greenhouse under long-day conditions (16 h/8 h) and temperature 20 ± 2 °C during the day and 16 ± 2 °C during the night, and labelled as the main culm. The pots were randomly arranged and periodically moved around to avoid positional effects. The plants were watered when needed.

Briefly, the data were scored from the spike of all labelled main culm plants after harvest for central/lateral spikelet traits, separately as follows: (1) the percentage of unfilled spikelets “UF” (%) were evaluated. Then central and lateral kernels were separated and the following grain traits were scored using a MARVIN grain analyser (GTA Sensorik GmbH, Neubrandenburg, Germany): (2) kernel number “KN”, (3) kernel length “KLn”, (4) kernel width “KWd”, (5) kernel area “KA” (6) kernel weight “KW” per main spike in gram (g), and (7) thousand kernel weight “TKW”. Four labelled spikes from the main culm of each accession were checked to obtain the average for all traits used for GWAS analysis.

### 4.2. Phenotypic Data Analysis

The experiment was arranged as a completely randomized design with 4 biological replications (plants) per accession. The frequency distribution of all traits was visualized using a histogram. Analysis of variance (ANOVA) of the phenotypic data was performed, which included 254 *intermedium*-spike barley accessions. All statistical analyses in this study were conducted using R 3.5.3 (R Core Team 2018 [43], Vienna, Austria). Data transformations were performed where necessary to normalise the trait distributions.

### 4.3. GBS-SNP Genotyping

All *intermedium* barley accessions were genotyped using genotyping-by-sequencing (GBS). DNA was extracted according to Doyle and Doyle protocol [44]. GBS was performed following (Mascher et al. 2013) [45]. Adapters were trimmed from reads with cutadapt [46] (Mascher 2011). Trimmed reads were mapped to the barley reference genome [7] with BWA-MEM [47], converted to BAM format with SAMtools [48] and sorted with Novosort (Novocraft Technologies Sdn Bhd, Petaling Jaya, Selangor, Malaysia, http://www.novocraft.com/). Multi-sample variant calling was done with SAMtools [49] using the command “mpileup” with the parameters “–DV”. The VCF output file was filtered with an AWK script available from https://bitbucket.org/ipk_dg_public/vcf_filtering. Only biallelic SNPs were considered. SNPs with minor allele frequency (MAF) of less than 5% and missing data of more than 20% were filtered out. Finally, we used 8653 SNPs for GWAS analysis of 254 *intermedium*-spike barley accessions.

### 4.4. Population Structure and Kinship Analyses

The population structure of the panel was assessed using STRUCTURE software 2.3.4, Stanford, California, USA [50]. Numbers of hypothetical subpopulation ranging k = 1 to 10 were assessed in an admixture model using 10,000 burn-in followed by 10,000 recorded Markov chain iterations. To estimate the sampling variance (robustness) of population structure inference, 10 independent runs were carried out for each k. The output from STRUCTURE was analyzed in STRUCTURE HARVESTER [51]. The ΔK statistics based on the rate of change in the logarithm of the probability of likelihood [LnP(D)] value between successive k values [52] was used to predict the optimum number of subpopulations. Population structure was also investigated using principle component analysis (PCA) using the Genome Association and Prediction Integrated Tool (GAPIT) R package [53]. To determine the number of PCs to use in clustering and GWAS analysis, a scree plot was generated by plotting the percentage of variance explained by the first 10 PCs against the number of PCs. The resulting PC loading for each line were exported for the creation of the graph using the ggplot function of R [43]. A kinship matrix (K) was constructed using GAPIT by setting the ‘group. from’ and ‘group. to’ parameters equal to the population size and the ‘group. by’ parameter equal to one.

### 4.5. Genome-Wide Association Mapping Analysis and Candidate Gene Identification

Genome-wide association mapping was performed with the Genome Association and Prediction Integrated Tool (GAPIT) [53]. Several models were tested for the identification of marker-trait associations (MTAs). A naive model utilizing only genotypic and phenotypic data using the general linear model (GLM). The principle component (P) model using the first (PC1) and second (PC2) components that were selected based on generated scree plots, were included in the model as fixed-effect covariate to correct for population structure. The kinship matrix (K) model, the relatedness between individuals was calculated and included in the model as random-effect covariate. Finally, the model (P + K), both the population structure PCs and kinship matrix K were included in mixed liner model (MLM) as fixed and random-effect covariates, respectively [54].

The optimal model (P + K) was selected based on the quantile-quantile (Q-Q) plots of *p-*values comparing the uniform distribution of the expected −log_10_ (*p*) to the observed −log_10_ (*p*) of all the evaluated traits (Supplementary Materials) and genome-wide association mapping was performed with the identified model. SNP sites with the lowest *p* value in the peak region (*p* < 10^−4^) were considered highly significant SNPs for phenotypic variation. We calculated the false discovery rate (FDR) at *p* value < 0.05 to determine the significance level of the SNP and exclude false-positive MTAs. Loci with a physical distance less than 100 bp were considered the same locus. Visualization of the significant SNPs was reported using Manhattan plots generated using the R package *qqman* [55]. The significant (FDR (*p*) < 0.05) or highly significant (FDR (*p*) < 0.01) identified associated markers were anchored physically using the recently published barley genome sequence [7]. Based on the SNPs physical position we isolate the candidate genes to be either contain the SNPs or the closest gene to the SNP position using the BARLEX IPK server (http://apex.ipk-gatersleben.de/apex/f?p=284:10).

## Figures and Tables

**Table 1:** Significant single-nucleotide polymorphisms (SNPs) showing the highest marker-trait associations for the central spikelet.

Traits	SNP ID	Chr	SNP Physical Position	Ref Allele	Alt Allele	Gene	HC/LC	Annotation
Kernel number “KN”	m1_556903593	1H	556903593	T	C	HORVU1Hr1G094980	HC_G	Early flowering 3 (ELF3)
	m7_168762979	7H	168762979	G	T	HORVU7Hr1G048880	HC_G	Sec14p-like phosphatidylinositol transfer family protein
	m7_168763009	7H	168763009	C	T	HORVU7Hr1G048880	HC_G	Sec14p-like phosphatidylinositol transfer family protein
% Unfilled spikelets “UF”	m2_35441939	2H	35441939	C	T	HORVU2Hr1G001640	HC_G	Mediator of RNA polymerase II transcription subunit 15a
	m7_109046266	7H	109046266	C	T	HORVU7Hr1G040320	LC_u	undescribed protein
Kernel weight “KW”	m1_556903593	1H	556903593	T	C	HORVU1Hr1G094980	HC_G	Early flowering 3
	m2_647258179	2H	647258179	A	G	HORVU2Hr1G091170	HC_G	Expansin B3
	m2_653986096		653986096	C	A	HORVU2Hr1G092530	HC_G	Aldehyde dehydrogenase family 3 member F1
Thousand kernel weight “TKW”	m2_727927744	2H	727927744	T	C	HORVU2Hr1G113260	HC_G	N-terminal nucleophile aminohydrolases (Ntn hydrolases) superfamily protein
	m2_727927750	2H	727927750	G	T	HORVU2Hr1G113260	HC_G	N-terminal nucleophile aminohydrolases (Ntn hydrolases) superfamily protein
	m2_727927781	2H	727927781	G	A	HORVU2Hr1G113260	HC_G	N-terminal nucleophile aminohydrolases (Ntn hydrolases) superfamily protein
	m3_661788536	3H	661788539	A	C	HORVU3Hr1G099530	HC_G	UDP-Glycosyltransferase superfamily protein
	m3_661788539	3H	661788539	T	G	HORVU3Hr1G099530	HC_G	UDP-Glycosyltransferase superfamily protein
	m3_661788540	3H	661788539	A	G	HORVU3Hr1G099530	HC_G	UDP-Glycosyltransferase superfamily protein
	m3_661788542	3H	661788539	A	T	HORVU3Hr1G099530	HC_G	UDP-Glycosyltransferase superfamily protein
Kernel area “KA”	m3_661788536	3H	661788539	A	C	HORVU3Hr1G099530	HC_G	UDP-Glycosyltransferase superfamily protein
	m3_661788539	3H	661788539	T	G	HORVU3Hr1G099530	HC_G	UDP-Glycosyltransferase superfamily protein
	m3_661788540	3H	661788539	A	G	HORVU3Hr1G099530	HC_G	UDP-Glycosyltransferase superfamily protein
	m3_661788542	3H	661788539	A	T	HORVU3Hr1G099530	HC_G	UDP-Glycosyltransferase superfamily protein
	m4_557589531	4H	557589531	C	T	HORVU4Hr1G067140	HC_G	Protein kinase superfamily protein
	m7_548419008	7H	548419008	G	T	HORVU7Hr1G090180	LC_u	undescribed protein
Kernel width “KWd”	m2_727927744	2H	727927744	T	C	HORVU2Hr1G113260	HC_G	N-terminal nucleophile aminohydrolases (Ntn hydrolases) superfamily protein
	m2_727927750	2H	727927750	G	T	HORVU2Hr1G113260	HC_G	N-terminal nucleophile aminohydrolases (Ntn hydrolases) superfamily protein
	m2_727927781	2H	727927781	G	A	HORVU2Hr1G113260	HC_G	N-terminal nucleophile aminohydrolases (Ntn hydrolases) superfamily protein
Kernel Length “KLn”	m7_548419008	7H	548419008	G	T	HORVU7Hr1G090180	LC_u	undescribed protein

**Table 2:** Significant SNPs showing the highest marker-trait associations for the lateral spikelet.

Traits	SNP ID	Chr	SNP Physical Position	Ref Allele	Alt Allele	Gene	HC/LC	Annotation
Kernel number “KN”	m7_168762979	7H	168762979	G	T	HORVU7Hr1G048880	HC_G	Sec14p-like phosphatidylinositol transfer family protein
	m7_168763009	7H	168763009	C	T	HORVU7Hr1G048880	HC_G	Sec14p-like phosphatidylinositol transfer family protein
	m7_651215909	7H	651215909	C	T	HORVU7Hr1G120030	HC_G	Delta(24)-sterol reductase
% Unfilled spikelets “UF”	m2_647258179	2H	647258179	A	G	HORVU2Hr1G091170	HC_G	Expansin B3
	m2_649489024	2H	649489024	G	A	HORVU2Hr1G091760	LC_TE	Retrotransposon protein, putative, unclassified
	m2_649557855	2H	649557855	T	C	HORVU2Hr1G091830	HC_G	undescribed protein
	m2_651372029	2H	651372029	G	A	HORVU2Hr1G092180	HC_G	Elongation factor G
	m2_653986096	2H	653986096	C	A	HORVU2Hr1G092530	HC_G	Aldehyde dehydrogenase family 3 member F1
	m2_654165703	2H	654165703	A	G	HORVU2Hr1G092600	HC_G	multiprotein bridging factor 1A
	m2_654165718	2H	654165718	C	G	HORVU2Hr1G092600	HC_G	multiprotein bridging factor 1A
	m2_654165739	2H	654165739	C	A	HORVU2Hr1G092600	HC_G	multiprotein bridging factor 1A
	m6_115087828	6H	115087828	G	T	HORVU6Hr1G028720	HC_G	V-type ATP synthase subunit D
Kernel weight “KW”	m7_168762979	7H	168762979	G	T	HORVU7Hr1G048880	HC_G	Sec14p-like phosphatidylinositol transfer family protein
	m7_168763009	7H	168763009	C	T	HORVU7Hr1G048880	HC_G	Sec14p-like phosphatidylinositol transfer family protein
	m7_651215909	7H	651215909	C	T	HORVU7Hr1G120030	HC_G	Delta(24)-sterol reductase
Thousand kernel weight “TKW”	m2_727927744	2H	727927744	T	C	HORVU2Hr1G113260	HC_G	N-terminal nucleophile aminohydrolases (Ntn hydrolases) superfamily protein
	m2_727927750	2H	727927750	G	T	HORVU2Hr1G113260	HC_G	N-terminal nucleophile aminohydrolases (Ntn hydrolases) superfamily protein
	m3_208358229	3H	208358229	C	A	HORVU3Hr1G036930	HC_G	Mediator of RNA polymerase II transcription subunit 27
	m4_26351423	4H	26351423	G	A	HORVU4Hr1G009300	HC_G	HXXXD-type acyl-transferase family protein
Kernel area “KA”	m2_8906731	2H	8906731	T	G	HORVU2Hr1G003720	HC_G	GDSL esterase/lipase
	m2_727927744	2H	727927744	T	C	HORVU2Hr1G113260	HC_G	N-terminal nucleophile aminohydrolases (Ntn hydrolases) superfamily protein
	m2_727927750	2H	727927750	G	T	HORVU2Hr1G113260	HC_G	N-terminal nucleophile aminohydrolases (Ntn hydrolases) superfamily protein
	m3_208358229	3H	208358229	C	A	HORVU3Hr1G036930	HC_G	Mediator of RNA polymerase II transcription subunit 27
	m7_548419008	7H	548419008	G	T	HORVU7Hr1G090180	LC_u	undescribed protein
Kernel width “KWd”	m2_727927744	2H	727927744	T	C	HORVU2Hr1G113260	HC_G	N-terminal nucleophile aminohydrolases (Ntn hydrolases) superfamily protein
	m2_727927750	2H	727927750	G	T	HORVU2Hr1G113260	HC_G	N-terminal nucleophile aminohydrolases (Ntn hydrolases) superfamily protein
	m3_208358229	3H	208358229	C	A	HORVU3Hr1G036930	HC_G	Mediator of RNA polymerase II transcription subunit 27
	m4_26351423	4H	26351423	G	A	HORVU4Hr1G009300	HC_G	HXXXD-type acyl-transferase family protein
Kernel Length “KLn”	m5_619972700	5H	619972700	A	G	HORVU5Hr1G104750	HC_G	Bifunctional inhibitor/lipid-transfer protein/seed storage 2S albumin superfamily protein|none

## Supplementary Materials

The following are available online at https://www.mdpi.com/2223-7747/9/12/1655/s1, Figure S1: Model-based subdivision of population structure. The plot shows the estimation of the number of populations by delta K value (ΔK)., Figure S2 The quantile-quantile (Q-Q) plots of p-values comparing the uniform distribution of the expected –log10 (p) to the observed –log10 (p) of all the evaluated traits., Table S1: Total number of *intermedium*-spike barley accessions and their origin., Table S2: The average number of SNPs/Mb for all chromosomes.
