# Supplementary material for: Dissecting the Genetic Basis of Lateral and Central Spikelet Development and Grain Traits in Intermedium-Spike Barley (Hordeum vulgare Convar. Intermedium)
Source: Plants (Basel). 2020 Nov 26;9(12):1655. doi: 10.3390/plants9121655 (PMC7760360; doi:10.3390/plants9121655)
Supplement: Supplementary file 1 [file plants-09-01655-s001.pdf]

Table S1. Total number of *intermedium*-spike barley accessions and their origin

Origin	Number of accessions
Asia	157
Africa	35
North America	26
Europe	21
Unknown	15
**Total**	254

Table S2. The average number of SNPs/Mb for all chromosomes

Chromosome	From (bp)	To (bp)	From (cM)	To (cM)	No. of SNPs	average of SNPs/cM
**Chr1**	51,895	558,222,570	0.106232	132.7432	987	7.4
**Chr2**	30,562	765,158,691	0.694519	149.1501	1,458	9.8
**Chr3**	694,165	698,895,996	0.032066	154.882	1,382	8.9
**Chr4**	54,127	646,290,210	0.637394	116.9763	905	7.7
**Chr5**	252,747	669,806,711	0	169.4083	1,431	8.4
**Chr6**	700,476	583,095,138	0.715831	126.4877	1,052	8.3
**Chr7**	176,267	656,941,572	0.212465	141.1676	1,438	10.2

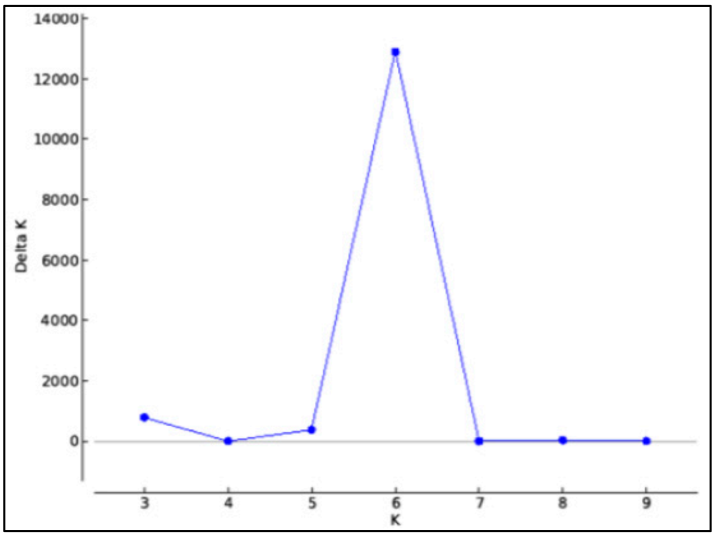

Figure S1 Model-based subdivision of population structure. The plot shows the estimation of the number of populations by delta K value (ΔK).

The number of subpopulation (K) was plotted against the ΔK calculated from Structure, and the peak of the broken line graph was observed at K=6 (Fig.), indicating that the population was basically divided into six subpopulations.

Q-Q plot KN (central spikelets)

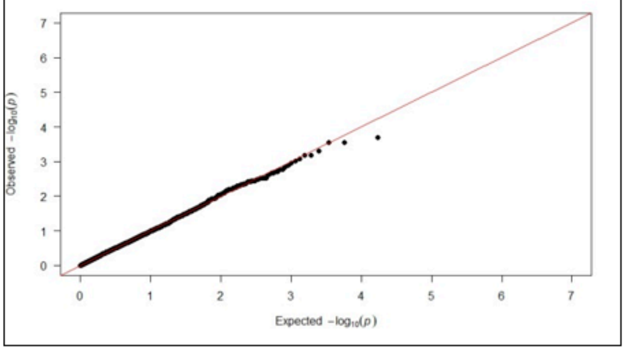

Q-Q plot KN (lateral spikelets)

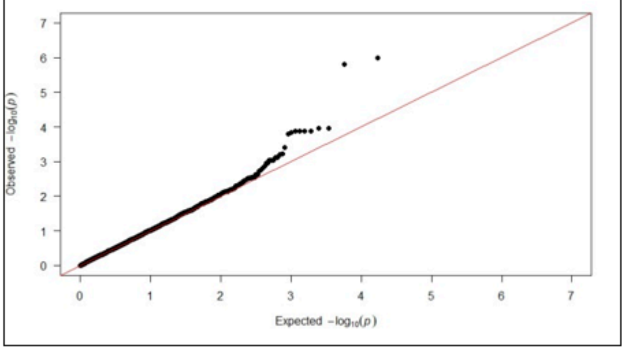

Q-Q plot UF % (central spikelets)

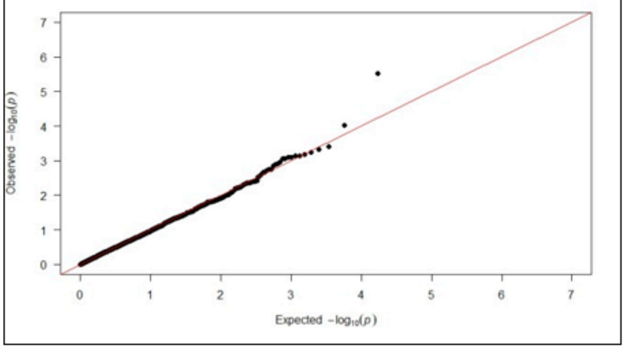

Q-Q plot UF % (lateral spikelets)

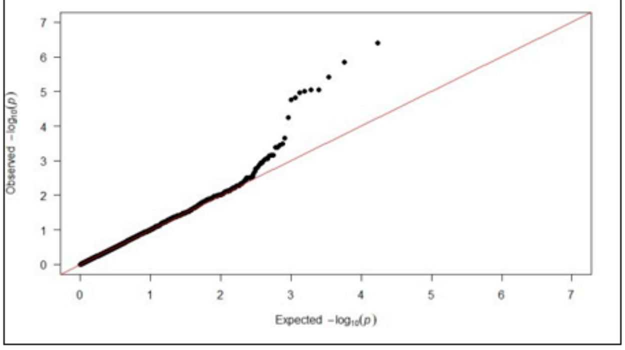

Q-Q plot KW (central spikelets)

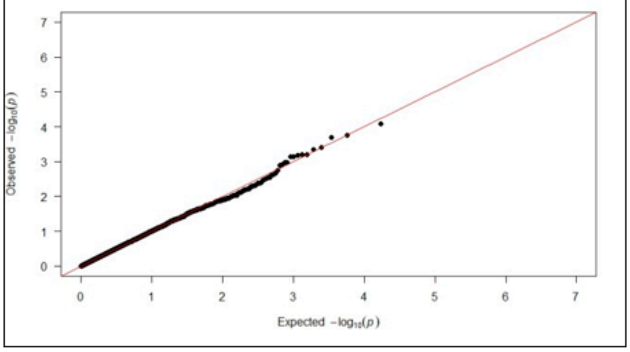

Q-Q plot KW (lateral spikelets)

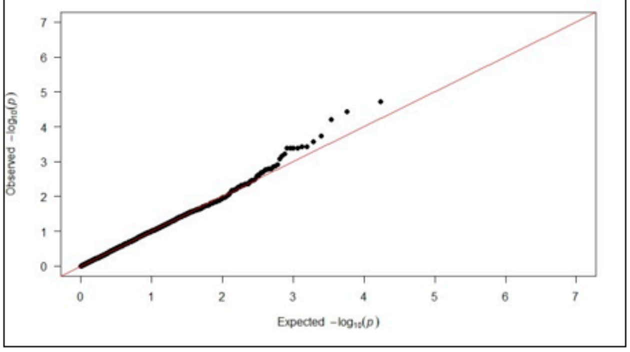

Q-Q plot TKW (central spikelets)

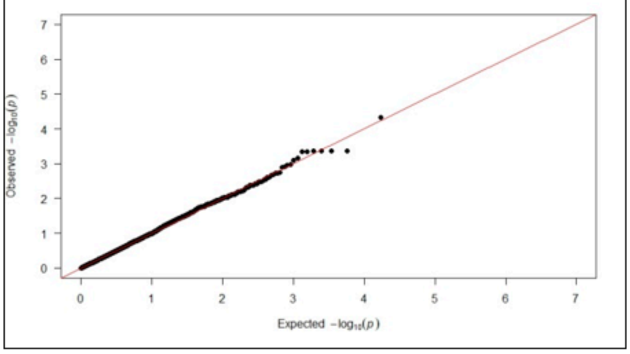

Q-Q plot TKW (lateral spikelets)

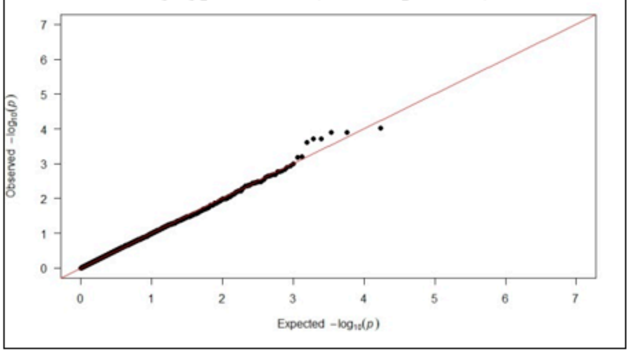

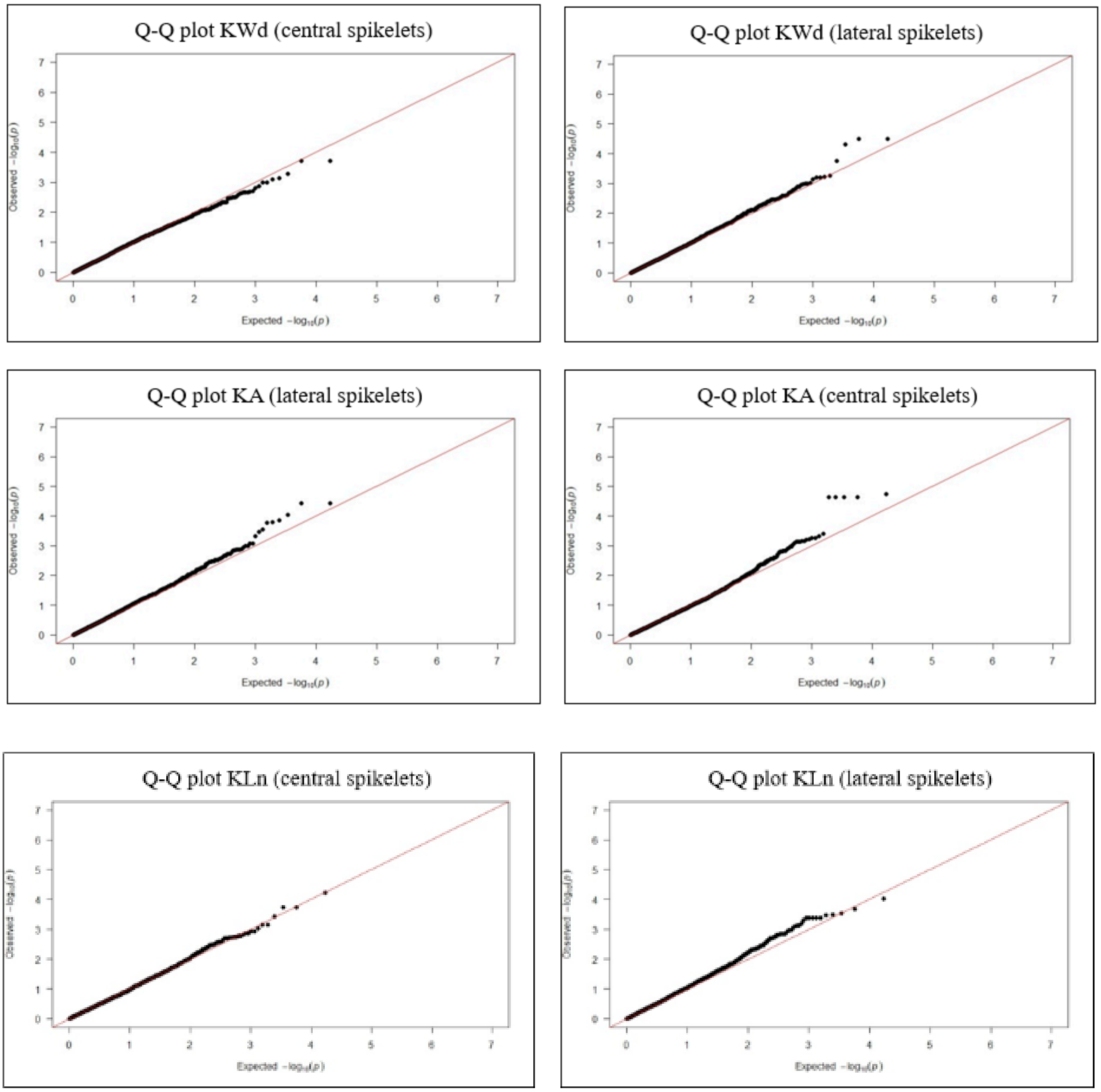

Figure S2 The quantile-quantile (Q-Q) plots of *p*-values comparing the uniform distribution of the expected $-\log_{10}(p)$ to the observed $-\log_{10}(p)$ of all the evaluated traits
